# Epidemiology of intestinal helminthiases in a rural community of Ethiopia: Is it time to expand control programs to include *Strongyloides stercoralis* and the entire community?

**DOI:** 10.1371/journal.pntd.0008315

**Published:** 2020-06-04

**Authors:** Aranzazu Amor Aramendia, Melaku Anegagrie, Derjew Zewdie, Elena Dacal, Jose M. Saugar, Zaida Herrador, Tadesse Hailu, Mulat Yimer, María V. Periago, Esperanza Rodriguez, Agustín Benito

**Affiliations:** 1 Mundo Sano Foundation, Madrid, Spain; 2 National Center for Tropical Medicine, Institute of Health Carlos III, Madrid, Spain; 3 Department of Microbiology, Immunology and Parasitology, College of Medicine and Health Science, Bahir Dar University, Bahir Dar, Ethiopia; 4 Laboratory of Reference and Research on Parasitology, National Centre for Microbiology, Institute of Health Carlos III, Madrid, Spain; 5 Mundo Sano Foundation, Argentina; 6 National Scientific and Technical Research Council (CONICET), Buenos Aires, Argentina; University of California, UNITED STATES

## Abstract

**Background:**

Soil transmitted helminths are highly prevalent worldwide. Globally, approximately 1.5 billion people are infected with *Ascaris lumbricoides*, *Trichuris trichiura* or hookworm. Endemic countries carry out periodic mass treatment of at-risk populations with albendazole or mebendazole as a control measure. Most prevalence studies have focused on school aged children and therefore control programs are implemented at school level, not at community level. In this study, the prevalence of intestinal helminths, including *Strongyloides stercoralis*, was examined using a comprehensive laboratory approach in a community in north-western Ethiopia.

**Methods:**

A cross-sectional survey was conducted on 792 individuals ≥5 years old in randomly selected houses in a rural district. Stools were examined using three techniques: a formol-ether concentration, the Baermann technique and a real time polymerase chain reaction test (these last two specific for *S*. *stercoralis)*. Statistical analyses were performed between two large age groups, children (≤14 years old) and adults (≥15 years old).

**Results:**

The prevalence of helminths was 91.3%; (95% CI: 89.3–93.3%). Hookworm was the most prevalent, 78.7% (95% CI 75.6–81.4%), followed by *S*. *stercoralis* 55.7% (95% CI 52.2–59.1%). Co-infection with both was detected in 45.4% (95% CI 42.0–49.0%) of the participants. The mean age of hookworm-infected individuals was significantly higher than non-infected ones (p = 0.003). Also, *S*. *stercoralis* infection was significantly associated with age, being more prevalent in adults (p = 0.002).

**Conclusions:**

This is the highest prevalence of *S*. *stercoralis* detected in Ethiopia so far. Our results highlight the need of searching specifically for infection by this parasite since it usually goes unnoticed if helminth studies rely only on conventional diagnostic techniques, i.e. Kato-Katz. Moreover, the focus of these programs on children undermines the actual prevalence of hookworm. The adult population acts as a reservoir for both hookworm and *S*. *stercoralis* and this fact may negatively impact the current control programs in Ethiopia which only target treatment of school aged children. This reservoir, together with a lack of adequate water, sanitation and hygiene, increases the probability of re-infection in children. Finally, the high prevalence of *S*. *stercoralis* found calls for a comprehensive diagnostic approach in endemic areas in addition to a revision of control measures that is, adding ivermectin to current albendazole/mebendazole, since it is the drug of choice for *S*. *stercoralis*.

## Author summary

Soil-transmitted-helminthiases and schistosomiasis have major programs for control within the neglected tropical diseases agenda. Deworming programs, that is, periodic mass drug administration, with albendazole or mebendazole and praziquantel, for at-risk populations, are the main strategy for control and success in interrupting the transmission of these diseases. But despite huge efforts made, programs are still unsuccessful in controlling STH in endemic areas in Africa. In Ethiopia most parts of the country are suitable for helminth transmission. Mapping for identification of risk areas for hookworm, *A*. *lumbricoides*, *T*. *trichiura* and *Schistosoma* spp., has been conducted by the government, in order to prioritize the health agenda. The focus of the interventions are school- aged children. Another species of soil-transmitted helminth, *Strongyloides stercoralis*, of high morbidity, which requires special laboratory methods for diagnosis and a different treatment drug, is highly underestimated and not included in control programs. With the aim of knowing the prevalence of *S*. *stercoralis* and other intestinal helminths at the community level, a population in the rural area of north-western Ethiopia was studied using parasitological and molecular techniques. A high prevalence of helminths at the community level and specifically a high prevalence of *S*. *stercoralis* were detected. These results suggest there is a need to revise control programs in order to be able to cut transmission and to ensure universal health coverage that “leaves no one behind".

## Introduction

Infections by soil transmitted helminths (STHs) are among the most common worldwide: according to the World Health Organization (WHO), 820 million people are infected with roundworms (*Ascaris lumbricoides*), 460 million with whipworms (*Trichuris trichiura*) and 440 million with hookworm (*Ancylostoma duodenale* and *Necator americanus*) [1]. These parasites are transmitted by eggs released in human faeces that contaminate the soil in areas where sanitation is poor [2]. Although these four species have certain individual characteristics, they are grouped together in control programs, which are based on periodic mass drug administration (MDA) of at-risk population (preschool and school-aged children–PSAC and SAC–as well as women of childbearing age—WRA) with albendazole (ALB) or mebendazole (MEB) [3]. STHs are widely distributed in sub-Saharan Africa (SSA), with Ethiopia being one of the countries with the largest burden [4]. More than 80% of the population, of above 107 million, is estimated to be living in rural areas endemic for STHs [5, 6]. Accordingly, the country has launched different MDA interventions in recent years. The current policy for NTDs establishes the integration of STH and schistosomiasis control programs [7]. Hookworms, *A*. *lumbricoides* and *T*. *trichiura* are included in the group of neglected tropical diseases (NTDs) but a fifth species, *Strongyloides stercoralis*, has not been included because different diagnostic techniques (of good specificity but not enough sensitivity) and another drug, (IVM) rather than ALB, is required for its correct management [8]. Recently, there is growing awareness on the actual global burden of *S*. *stercoralis* [9, 10] and, although it is still underestimated, the WHO now mentions it as part of the STHs [11]. However, correct diagnosis and treatment of *S*. *stercoralis* prevalence of 21% was found in children aged 7 up to 10 years old using a combination of specific techniques [12]. Since this parasite has the ability of living for a long time in the human host [13], we hypothesized that prevalence in adults will be higher than prevalence in children. The objective of the current study was to determine the prevalence of *S*. *stercoralis*, by using a combination of techniques, at the community level, in a rural area with a known high prevalence of *S*. *stercoralis* in SAC.

## Methods

### Study area and population

**Fig 1:**
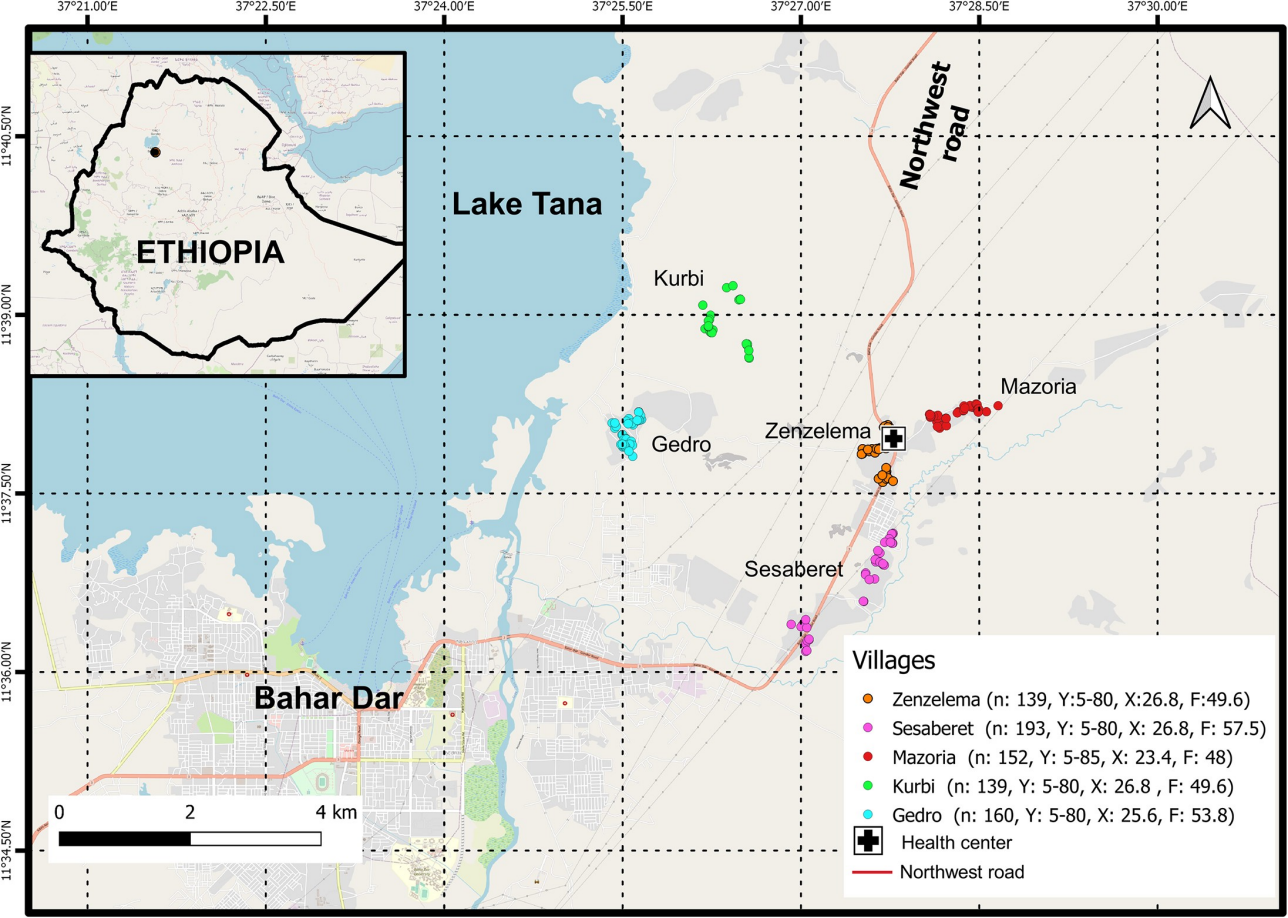
Area of the of the study, Northwestern Ethiopia: A district in the rural area of Bahir Dar city, the capital of the Amhara Regional State, in the south shore of Lake Tana. The five villages included in the study are showed. Data about the studied population, age and gender are noted. n = number of population recruited in the village Y = Range of age; $\bar{X}$ = mean of age; F = female (%) (Map: Sentinel-2 (ESA) image courtesy of the U.S. Geological Survey).

We conducted a cross-sectional study from February to June 2016, in a rural district (Zenzelema *kebele*) about 20 km east of Bahir Dar City, the capital of the Amhara Regional State (Fig 1). The district is located in the highlands of Ethiopia, at 1,700 to 2,000 m above sea level, and it is composed of nine villages, with a population of 11,130 (health center data). There are three distinct seasons: rainy season, from June to September; spring, from October until January; and a dry season the rest of the year. The main source of subsistence is farming, mostly cultivation of cereals and chat *(Catha edulis)*, livestock husbandry. Migratory flows are not common, but in recent years the population suffered displacements inside the villages due to government land management. The prevalence of STHs in the area is moderate (20% to 50%) and it is categorised as free of schistosomiasis [7]. School-based deworming interventions are launched once a year [14].

### Study design and sampling strategy

A list of population and houses was obtained from the registration books kept in the health center. In view of the main objective (to determine the prevalence of *S*. *stercoralis* at the community level), and because prevalence data for this species in the study area was unknown, a maximum prevalence of 50% was anticipated, with a marginal error of 5% and a 95% confidence interval. A design effect of 2 was taken into account, corresponding to the complex design. All inhabitants over five years of age living in the area continuously for at least six months, prior to the first visit of the work team, were included. A multistage cluster random sampling was implemented in two stages: first, for village selection, a probability sampling technique proportional to size was applied (five in total), second, a mean of 52 houses were randomly selected in each village. Further methodological explanations had been provided previously [12]. A total of 792 people, recruited in 241 houses, were included in the study. The study area lies between latitudes 11° 39’14.7” N and 11° 36’ 07.5” N and between longitudes 37° 26’ 25.7” E and 37° 28’ 39.5” E, at an altitude of 1,669–1,958 m above sea level. Mean distance to the health center was 2.3 Km. Fig 1 shows the geographical distribution of the houses across the district and the location of the district health center serving the population in the area.

### Ethics statement

The Amhara National Regional State Health Bureau Ethics Review Committee revised and approved the study protocol (Ref. n°: 1/87/2008). According to the principles of the Helsinki Declaration, informed consent for stool and blood examinations was sought, as well as withdrawal guarantee, guarantee of anonymity, treatment and follow up. Written informed consent was set for adults and children; for the second group, parents or guardians signed the consent form. Permits for exporting DNA for molecular analyses in Spain, were obtained from the Ethiopian Biodiversity Institute in Addis Ababa (Ref. n°: EBI71/671/2016). Participants were treated according to the Ethiopia Standard Treatment Guidelines [15]. All inhabitants from household included in the study were offered a single dose of ALB (400 mg) during the household visit, regardless of participation. The Amhara National Regional State Health Bureau kindly donated IVM for treatment of *S*. *stercoralis* infection.

### Sample collection

Every participant was asked to provide a single stool and blood sample at the same time. Stool samples were collected in a labelled container, 60 mL capacity. Before collection, participants received clear explanations about the required amount, that is, to completely fill the container; the sample was discarded if the patient was not able to follow instructions. Stool samples were sent to the laboratory at room temperature, without any preservation method, and processed within a maximum of four hours after collection. Approximately 600 microliters of venous blood samples were collected with the Multivette 600 EDTA Blood collection system and sent to the laboratory in a refrigerated container (4°C).

### Parasitological examination

The laboratory of Bahir Dar University was the sentinel site for parasitological analysis. A total of three techniques were used. 1) Formol-Ether Concentration (FEC). The sensitivity of this technique for egg detection has been pointed out in other studies based on a single sample [16]. Briefly, 0.5 gram (g) of stool were processed using the Bioparaprep MINI system (Leti Diagnostics, Barcelona, Spain) according to the manufacturer instructions; 2) Baermann Technique (BT): A total of30 g of stool were incubated at 26°C for 18 h with activated charcoal. After incubation, the samples was placed on a funnel with water at 37°C and left standing for one hour before the water from the bottom was collected and centrifuged at 2,000 rpm for 5 minutes [12]. *Strongyloides stercoralis* larvae were identified by the buccal cavity and the genital organs in rhabditiform larvae, and the posterior tip in filariform larvae; and 3) McMaster (MM) counting method: Briefly, 2 g of stool were suspended in 30 ml of saturated salt solution and mixed properly. Then, 0.5 ml aliquots were added to each of the two chambers of the MM slides, which were examined within 30–60 min of preparation. The total number of egg per gram (EPG) of each helminth species (except *S*. *stercoralis*, which is not detected by the MM technique) was obtained by multiplying the sum of eggs by 50. The intensity of infection was categorized as light, moderate or heavy [17]. Even though repeated sampling will increase the detection of parasites, mainly for *S*. *stercoralis*, the difficulties for accessing the area, which would extend too much the time of sample collection, were the main reason for examining only one sample. Nevertheless, for every technique, all the samples were replicated at least three times and examined by the three different microscopists.

### DNA amplification

PCR was performed only for *S*. *stercoralis* detection. One gram of stool sample was used for DNA extraction, after a concentration using the Bioparaprep MINI system with saline solution (0.9%), by using the QIAamp DNA stool mini kit (Qiagen, Hilden, Germany), following the manufacturer instructions. The DNA was sent to Madrid, Spain, in order to perform a qualitative real-time polymerase chain reaction (RT-PCR) assay, using a SybrGreen format (Invitrogen, San Diego CA, USA), as described by Saugar *et al*. [18]. *Strongyloides stercoralis*-specific primers targeting the 18S ribosomal subunit, as described by Verweij et al. [19] were used (Primer F: 5'-GAA TTC CAA GTA AAC GTA AGT CAT TAG C-3'; Primer R: 5'-TGC CTC TGG ATA TTG CTC AGT TC-3'). Purified genomic DNA from *Strongyloides venezuelensis* L3 was used as a positive control. The samples were assayed in duplicate. A third sample including 10 ng of *S*. *venezuelensis* DNA was also included as an internal inhibition control. No template controls were included in each run.

### Blood test

An Abbot Cell Dyn 1800 Haematology Analyzer (Abbott Diagnostics, Chicago, Illinois, United States) was used for blood counts (white blood cells, red blood cells and platelet). For haemoglobin (HGB) concentration, the adjustments for individuals living at altitudes higher than 1000 m above sea level were applied [20]. Blood samples were processed in the laboratory of the Amhara Public Health Institute.

### Data and definitions

Gender and age of the participants were recorded. For analyses, the participants were categorized as children (≤14 years old) and adults (≥15 years old). A Garmin Dakota 20 GPS was used for determining the location of every house included in the study (latitude, longitude and elevation). Infection by any helminth was considered positive when it was detected by any of the parasitological test used. For *S*. *stercoralis*, infection was noted as positive when at least one of the three diagnostic techniques was positive, i.e. presence of larvae in one/both parasitological test and or positive by PCR. Participants were diagnosed as co-infected when different parasites were identified in the sample by the different techniques, either parasitological or molecular, e.g.: hookworm in the FEC and S. *stercoralis* in PCR; hookworm in McMaster and *S*. *stercoralis* in BT.

### Statistical analysis

A descriptive analysis of parasitic infections was performed using frequency tables. Proportion and 95% confidence interval (CI) were used for the categorical variables while mean and standard deviation (SD) were used for the quantitative variables. Differences in parasite prevalence were assessed by the Chi-square test or Fisher’s exact test, and the association between infection and quantitative variables was analyzed by the *t*-test. The level of statistical significance was set at a value of *p* < 0.05. Statistical analyses were performed using the software package SPSS v20.0.

## Results

Mean age of participants was 24.4 (SD 16.64), ranging from 5 up to 85; 62% (95% CI 58.6–65.4%) of participants were adults (95% CI 58.6–65.4%) and 43.9% were male (95% CI 40.3–47.3%). The mean of HGB level, once adjusted by altitude, was 12.25 (SD 1.53), ranging from 3.1 to 19.8. Table 1 summarizes the analytical results of the blood analyses. The mean HGB in the group of children was significantly lower than in the group of adults (p < 0.01). Mean corpuscular haemoglobin was also significantly lower in children (p < 0.01).

**Table 1:** Blood values in the studied population: Percentage with normal, low and high analytical values. (WBC: White blood cell; LYMPH: Lymphocytes; MID: Mid cells total count; NEUTROPH: Neutrophils; HCT: Hematocrit; HGB: Haemoglobin).

Normal values	LOW (%)	NORMAL (%)	HIGH (%)
WHITE BLOOD COUNT (3–10.2 x 10^3^/L)	0.1	66.3	33.6
LYMPHOCYTES (0.8–3.2 x 10^9^/L)	_	46.5	53.5
LYMPHOCYTES % (14–46)	0.3	64.4	35.4
MID* CELLS (0.1–24%)	_	96.2	3.8
NEUTROPHILES (2.4–6.4 x 10^9^/L)	18.7	70.3	11
NEUTROPHILES % (37–80)	36.4	63.6	_
*HEMATOCRIT* (36–54%)	3.3	95.5	98.5
HAEMOGLOBINE (14–18 g/dL)	78	21.3	0.7
PLATELET (98–350 x 10^3^/L)	5.4	72.8	21.8

* Monocytes, eosinophils, basophils

### Helminth infections

The prevalence of any intestinal helminth infection was 91.3% (95% CI) 89.3–93.3%), with hookworm being the most prevalent, 78.7% (95% CI 75.6–81.4%). *S*. *stercoralis* was the second most prevalent 55.68% (95% CI 52.2–59.1%), and *S*. *mansoni* was detected in 48 patients (6.1%; 95% CI 4.2–7.6%). Other helminths included *A*. *lumbricoides* (2.5%; 95% CI 1.3–3.5%), *Hymenolepis nana* (1.6%; 95% CI 0.7–2.5%), *Enterobius vermicularis* (1.3%; 95% CI 0.3–2%), *T*. *trichiura* (0.6%; 95% CI 0.09–1.1%) and one case of *Fasciola* spp. (0.1%; 95% CI 0.09–0.3%). Half of the positive patients (50.6%; 95% CI 45.9–52.9%) were infected with two and up to four different helminth species, being hookworm with *S*. *stercoralis* the most frequent co-infection, as they were detected together in the 45.4% (95% CI 42–49%) of the participants.

**Fig 2:**
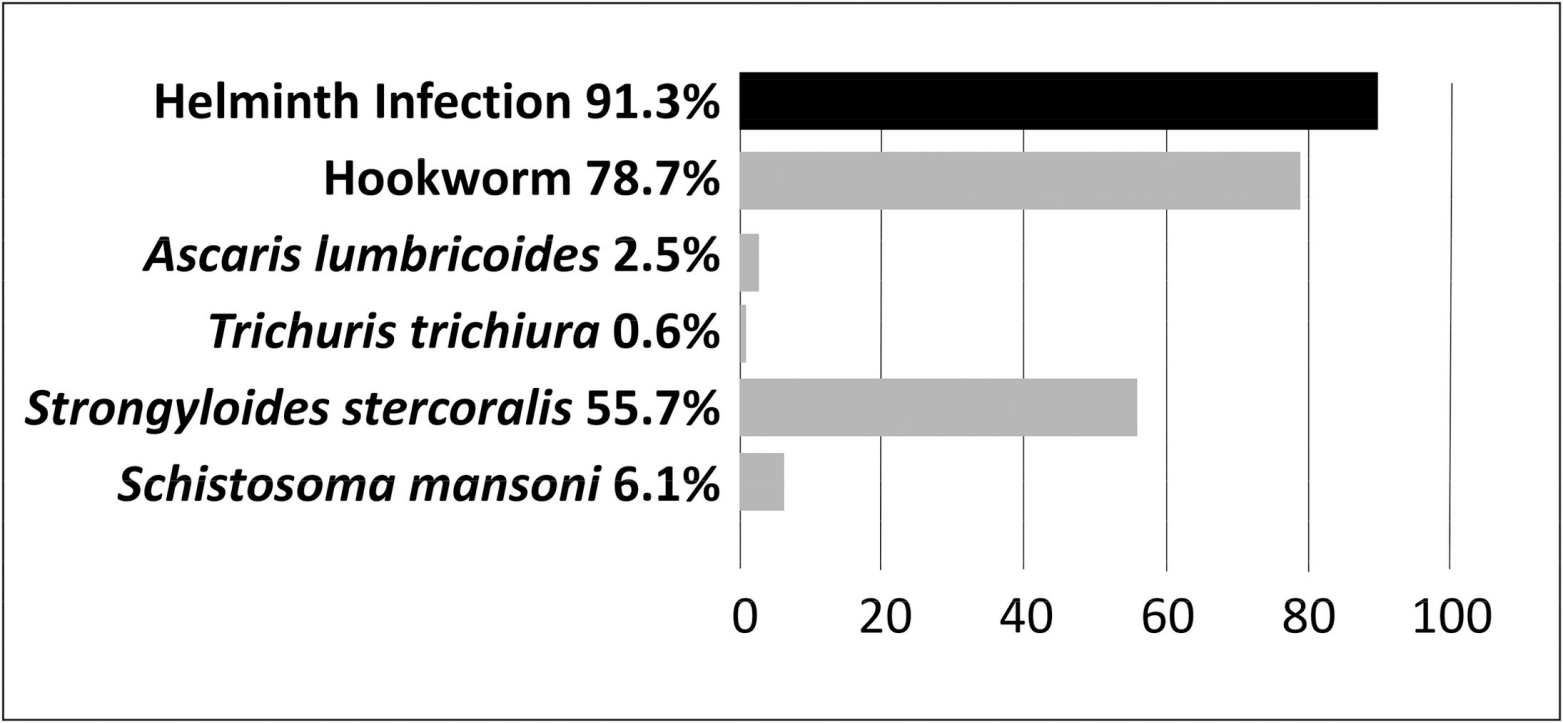
Helminth infections in the 792 participants. In the top, (black) the overall helminth prevalence. At the bottom (grey colour) prevalence of the soil transmitted helminths, (including *Strongyloides stercoralis*), *Schistosoma mansoni*, and other minor findings mentioned in the text.

**Fig 3:**
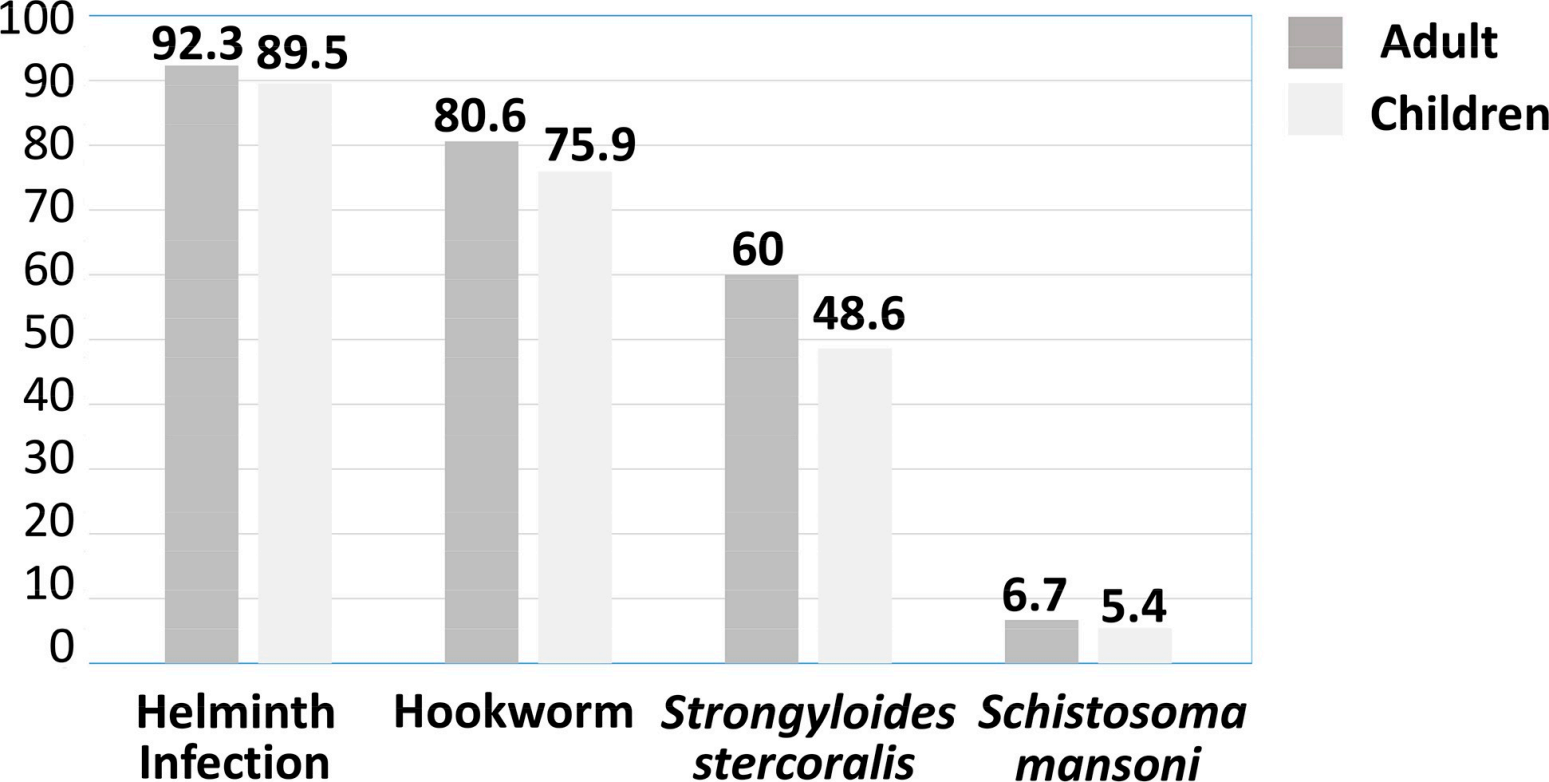
Overall helminth prevalence and prevalence of the soil transmitted helminths, (including *Strongyloides stercoralis*) and *Schistosoma mansoni* in the groups of adults and children.

The distribution of helminths by either gender or age was not different neither in the global sample nor between the two age categories (adults and children). The distribution of helminth infections among children was 89.5% (95% CI 85–93%), lower than among adults 92.3%; (95% CI 90–94.7%); this difference was not statistically significant. Nevertheless, the mean age of infected participants was significantly higher than the mean age of non-infected ones (p = 0.02). (See Fig 2 and Fig 3)

### Hookworm infections

The prevalence of 78.7% for hookworm was a result of the combination of the FEC (64.3%; 95% CI 60.77–67.5%) and MM (75.2%; 95% CI 71.1–77.4%) techniques; 27 patients (3.4%; 95% CI 2.1–4.7%) were only diagnosed by FEC, while 113 participants (14.3%; 95% CI 11.8–16.7%) were detected solely by the MM technique, which was a more sensitive technique for hookworm detection in our sample (p<0.01). The mean EPG was 445 (range: 50–6,600); the vast majority of infections were of low intensity (96.1%; 95% CI 95–97.7%). No association was found between gender and hookworm infection; also, no difference was found between adults vs. children groups. Nonetheless, the mean age of hookworm-infected participants was significantly higher than the non-infected ones (p < 0.01) (Fig 3). No relationship was found between altitude and hookworm infection but an association was found with both latitude (p < 0.01) and longitude, (p = 0.02), that is, hookworms were more prevalent in areas closer to the lake. Also, hookworm infection was significantly higher in the samples collected during the rainy season (p = 0.03). No association between HGB levels and hookworm infection was found in our sample, although lower lymphocyte absolute counts and percentages were associated with hookworm infection (p < 0.01 and p = 0.02, respectively).

### *Strongyloides stercoralis* infections

**Fig 4:**
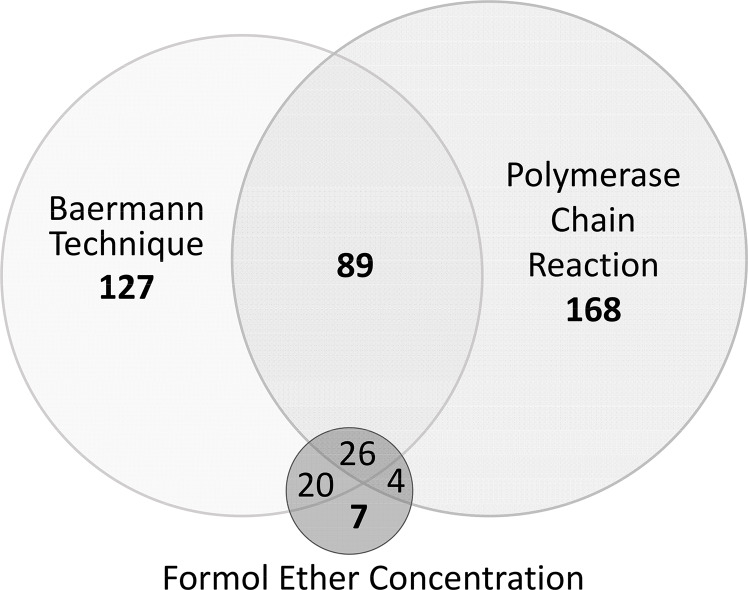
Venn diagram comparing *Strongyloides stercoralis* diagnosis of the 441 positive results by the Baermann, Formol Ether Concentration and Polymerase Chain Reaction techniques.

The prevalence of *S*. *stercoralis* was 55.7% (95% CI 52.2–59.1%) as a result of the combination of techniques: 34.3% (95% CI 31–37.7%) were positive by parasitological techniques, 7%, (95% CI 5.3–8.9%) by FEC, 33% by BT (95% CI 30–36.3%) and 36.2% of the samples were positive by PCR (95% CI 33.1–39.8%). However, not all samples positive by parasitological techniques were found to be positive using PCR, i.e. *S*. *stercoralis* DNA was detected in 287/441 (65.1%; 95% CI 60.6–70%) of the total of *S*. *stercoralis* infected, but only 119 of the 272 samples positive by parasitology (43.8%; 95% CI 38–50%) were confirmed by PCR and there were 168 of the total 441 positive for *S*. *stercoralis* (38.1%; 95% CI 33.56–42.63%) that were diagnosed only by PCR. On the other hand, 154/441 (34%; 95% CI 30.06–38.9%) were diagnosed by parasitological techniques alone. Fig 4 shows the prevalence of *S*. *stercoralis* in the 441 samples, as determined by individual techniques and by the combination of all three. The PCR technique showed higher sensitivity than the parasitological techniques (p < 0.01). *Strongyloides stercoralis* infection was significantly more prevalent in the group of adults (p < 0.01) and the mean age of *S*. *stercoralis*-infected people was significantly higher than the mean of non-infected ones (p < 0.01) (Fig 3). There was no association found with gender and infection with *S*. *stercoralis*. The prevalence in the villages ranged from 50.7% up to 61.9%, but no difference was noticed between them. *S*. *stercoralis* was detected in lower altitudes (p = 0.08). The platelet count showed to be lower in patients with infection (p = 0.06).

### *Schistosoma mansoni* infection

The prevalence of *S*. *mansoni* infection was 6.5% and it was higher in adults (6.7%; 95% CI 4.4–8.9%) than children (5.5%; 95% CI 2.8–8%), but the difference was not statistically significant. Infection with this helminth was significantly higher in the samples collected during the rainy season (p < 0.01) and in patients living more to the east, as well as in higher altitude (p < 0.01).

## Discussion

The goal of this study was to assess the prevalence of intestinal helminths, including *S*. *stercoralis*, at the community level, in an area previously found to be of high prevalence for SAC [12]. The prevalence of helminths, including *S*. *stercoralis*, was very high in the population. Based on our results, we would like to raise the issue of the implementation of additional diagnostic methods for *S*. *stercoralis* in areas of high prevalence of hookworm. Furthermore, for control programs, the appropriateness of taking into account this parasite, as it requires a different treatment. Moreover, given that such programs exclude the adult population, we would like to open the discussion on the possibility of its inclusion. Overall, the study reproduces the prevalence of STH in Africa, that is, hookworm is widely distributed in rural areas [21], but the prevalence of STH in our population is remarkably high. This fact is related to the high sensitivity of the FEC for egg detection [16]. The results obtained will be of great interest for surveillance studies and control policies in the country. Data of the STH prevalence from SAC-based surveys in Amhara region in recent years, showed a prevalence ranging from 36.4% [22], up to 66.4% [23]; only a small percentage of children had high intensity infections, as found in the population from the current study. A study conducted in the southwestern part of the country, in an area of similar altitude and at community level, showed an STH prevalence of 52.1%, including infections with both protozoans and helminths [24]. Again the majority of STH infections were categorized as light; the distribution of hookworm by age was similar to that found in the current study.

The results herein point out that, frequently, hookworm infection is concentrated in adult populations and so SAC-MDA based programs miss an important reservoir that will challenge the success of control programs [25]. On the other hand, as the intensity of infection is light, as it is in most of the studies; the fast processing of the samples that was performed (a few hours after collection) but, above all, the laboratory procedures used (concentration of the sample and combination of techniques), could probably explain the highest prevalence found. On the other hand, with regards to the haematologic counts found in hookworm infected individuals, the lower lymphocyte count will be probably related to a malnutrition status, as already described [26].

To our knowledge, this is the first study carried out at community level specifically for the detection *S*. *stercoralis* in Ethiopia and this is the highest prevalence ever detected at the community level in the country. A very recent review of studies published in the last ten years on strongyloidiasis in Ethiopia [27] showed, a mean prevalence of 2.5% in healthy adults and 11% in HIV positive adults. In Africa, only a study carried out in Gabon showed a prevalence higher than our study, 91.8%, but it was in a population of 15 PSAC [28, 29]. There are well known highly endemic communities in Australia, where remote communities showed a prevalence of >60% [30], Cambodia, of almost 45% [31]. In Argentina, a higher prevalence (76%) was detected at community level [32], in Bangladesh 61% prevalence [33] and in African migrants from Sudan and Somalia, 46% and 23% prevalence, respectively [34] when using serology for diagnosis. The high prevalence in our study is due to the combination of two specific, parasitological and molecular techniques; while each test identified a similar proportion of infections (33.0% and 36.2%), only 115 out of 441 samples (26%; 95% CI 22.8–29.2%) were positive for both assays. This pattern is almost the same as the one detected in a previous study focused on SAC [12] and in other similar studies [35]. In the above mentioned revision in Ethiopia, the revised studies were based solely on microscopy techniques, which probably resulted in lower prevalence due to the higher sensitivity of molecular techniques. Even though data on the combination of techniques are not evaluated in this paper, the authors point out the fact that strongyloidiasis is underestimated Ethiopia and that there is a need to approach this issue through the use of a combination of diagnostic methods. [27]. The co-infection of hookworm and *S*. *stercoralis* in our sample was as expected: there is evidence in different geographic locations of similar patterns, as both parasites share the same transmission pathway [36, 37]. Moreover, the underestimation of *S*. *stercoralis* in areas of high prevalence of hookworm should be considered since if specific techniques are not used, it will not be detected. In the frame of a growing awareness of non-communicable diseases in developing countries, if the prescription of corticosteroids increases as expected, the lack of awareness of infection by *S*. *stercoralis* will lead to a growing challenge for clinicians [38]. Finally, the association between lower platelet counts and *S*. *stercoralis* infection found in the current study has not previously been documented; further research would be interesting in in order to know if there is an association between this parameter and the clinical expression of the infection.

With respect to the other helminths found in this study, *S*. *mansoni* is known to be common in highland Ethiopia [7] around Lake Tana. Displacement towards small rivers in the rainy season, following the water flow, has been described [39] and could explain the higher prevalence found in eastern locations from our sample. However, it is important to point out the finding of 6.5% prevalence of *S*. *mansoni* since the study area has been classified as free of schistosomiasis in the last mapping carried out by the government [7]. Low *S*. *mansoni* egg counts require a more sensitive technique than the ones used in the current study; even more, the FEC could damage the *S*. *mansoni* eggs, as the processing includes a centrifugation step, therefore, the real prevalence in the study population may have been underestimated and will probably be higher when using a more sensitive and specific technique [40, 41]. Finally, the occurrence of *Fasciola* spp. has previously been described in human being in the area of study [42]. Due to the morbidity associated to the infection, more studies are needed to know the actual burden of fascioliasis, as there is no available treatment in the country, and for planning public health measures involving also veterinary authorities.

Some limitations of the study must be pointed out. First of all, the use of single stool samples lowers the sensitivity of coprological techniques and therefore the prevalence of some of the parasites found could indeed be higher [43]. Unfortunately, when working in remote rural areas, it is difficult to obtain more than one sample. Although serology was not used to detect infection by *S*. *stercoralis*, the use of both microscopy and molecular biology techniques, has allowed to detect an area with high prevalence. Maybe, a higher prevalence would be observed if serological tests were used, but the advantage of using direct methods certifies that they are active infections.

Helminth infections are of global importance and efforts for reducing the morbidity associated with them in endemic areas focus on PSAC, SAC and women of childbearing age. Those programs would not be expected to have an impact on the transmission, as they neglect adult populations; also, mapping in endemic areas could underestimate the actual STH prevalence, mostly for *S*. *stercoralis* infection, if only a single traditional coprological technique is used (i.e. direct smear or Kato-Katz); this would have a direct effect on public health decisions. Based on the results of the current study, for both, short-term and long-term programs, as well as for monitoring interventions, authorities must consider: 1) reviewing diagnostic approaches in order to be able to detect low helminth infections and specifically infections by *S*. *stercoralis*, 2) incorporating IVM alongside ALB/MEB in MDA programs in order to be able to lower the burden of infection for all five species of STHs, and 3) expanding MDA programs at community level in areas of high endemicity of hookworm and *S*. *stercoralis* given the high prevalence found in the adult population that acts as an important reservoir for these parasites, thus hindering the success of control programs.

As control programs progress, the intensity of infection by STH will be light. In some areas of the country, in the absence of a comprehensive protocol, this will result in an underestimation of the actual STH prevalence; in the same way, in areas of high prevalence of hookworm, infection by *S*. *stercoralis* seems to be underestimated. For an integral control it will be advisable to include adult populations in control campaigns, and to administer IVM at the same time as ALB in MDA campaigns taking into account that *S*. *stercoralis-*hookworm coinfections are common.

## Supporting information

S1 Checklist STROBE checklist. (DOCX)
